# Impact of oocytes with CLCG on ICSI outcomes and their potential relation to pesticide exposure

**DOI:** 10.1186/s13048-017-0335-2

**Published:** 2017-07-10

**Authors:** Philippe Merviel, Rosalie Cabry, Karen Chardon, Elodie Haraux, Florence Scheffler, Naima-belhadri Mansouri, Aviva Devaux, Hikmat Chahine, Véronique Bach, Henri Copin, Moncef Benkhalifa

**Affiliations:** 1 Ob/Gyn Department, Regional University hospital, Morvan University, 29200 Brest, France; 2 ART and Reproductive Biology laboratory, University hospital and school of medicine, Picardie University Jules Verne, CHU Sud, 80054 Amiens, France; 3 PERITOX–INERIS laboratory, CURS, Picardie University Jules Verne, CHU Sud, 80054 Amiens, France; 4 Forte Bio et Unilabs France, 1 Rue Mozart, 92200 Clichy La Garenne, France

**Keywords:** Ovarian stimulation, Oocyte morphology, Dysmorphic cytoplasm, Pesticide exposure, ICSI outcomes

## Abstract

**Background:**

Oocyte quality is a key limiting factor in female fertility which is primarily reflected in morphological features. Centrally located cytoplasm granulation (CLCG) is one type of cytoplasmic dimorphism exhibited by oocytes that could be linked to pesticide exposure with a significant risk of decreased ICSI outcomes.

**Methods:**

This retrospective study included 633 women who were part of an intracytoplasmic spermatozoa injection (ICSI) program between 2009 and 2011. The participants lived in the Picardy region of France and had been exposed to pesticides. The participants were divided in two groups based on prevalence of oocytes with CLCG (LCLCG [*n* = 83]: low prevalence of oocytes with CLCG under 25%. HCLCG [*n* = 68]: high prevalence of CLCG over 75%). The embryological and clinical outcomes were analysed for both groups and were calculated using the difference between the two values.

**Results:**

Results for couples with HCLCG compared to LCLCG showed a decrease in embryo cleavage, ongoing pregnancy, and live birth rates (82%, 14%, 13% vs 99%, 32%, 30%, respectively).The early miscarriage rate was increased (47% vs 11%), with an OR of 3.1 (95%CI [2.1–4.1]). Due to high pesticide exposure (over 3000 g/ha), there is a higher risk of a resulting disturbed oocyte cohort with a high prevalence of CLCG over 75%.

**Conclusion:**

The high prevalence of oocytes with CLCG over 75% has a negative effect on embryos and the general ICSI clinical outcomes. Furthermore, a putative association between pesticide exposure and risk of CLCG was identified, justifying the need for further research and a potential need to find alternative assisted reproductive technologies for these couples.

**Trial registration:**

Tabacfertimasc. ID number: ID2011-A00634–37; registered 2011/2/8

## Background

The assessment of oocyte quality using an inverted microscope is mainly based on cytological and morphological criteria. However, this evaluation does not fully reflect the level of oocyte maturation competency, fecundability, or its ability to support early embryonic development. It is commonly accepted that a “good quality” oocyte at metaphase II (MII) stage presents a moderately granular vacuole-free cytoplasm, a thin perivitelline space, a non-fragmented round polar body, and a round, homogeneous, non-dense zona pellucida [1–3]. However, it has been reported that more than 50% of collected oocytes after controlled ovarian stimulation (COS) present one or more anomalies [4, 5], and generally 93% of patients undergoing assisted reproductive technologies (ART) have at least one abnormal oocyte [6]. Oocyte dysmorphism could be exhibited as the presence of an abnormally granular cytoplasm, vacuoles, refractile bodies, a wide perivitelline space, an abnormal zona pellucida, aggregations in the endoplasmic reticulum, or other anomalies [7]. Among these abnormalities, cytoplasmic granularity most commonly affects embryo development [6, 8–10].

Centrally located cytoplasmic granulation (CLCG) was first defined by Serhal et al. [8] as “clearly delimited central granulations that are denser than the adjacent cytoplasm”. Some authors suggest that there is a relationship between CLCG and smooth endoplasmic reticulum clusters (sERC) [11] or aggregates of tubular smooth endoplasmic reticulum (aSERT) [12] with mitochondrial disturbance. Furthermore, Otsuki [13] observed that numerous small refractile (lipofuscin) bodies were located within the CLCG, suggesting that these abnormalities may have a common origin. The biomolecular explanation for this phenomenon is still enigmatic. However, the most plausible hypothesis that could explain CLCG dysmorphism is cytoplasmic immaturity [14, 15] which would be responsible for embryonic aneuploidy production at 52–57% [14, 16] and having 50% of risk to occur it in early meiotic maturation [17, 18]. Moreover, the most affected oocytes by toxic environment exposure are those at the pre-meiotic maturation stage [14, 16].

Despite the lack of clear explanation involving the real mechanism of CLCG production in oocytes, the impact on ART outcomes cannot be ignored. As already reported by Serhal et al. [8], the exhibited CLCG by oocytes declined implantation rate from 10% to just 1% without any ongoing pregnancy. Rienzi et al. [6] could to evaluate therefore the oocyte morphology and conclude that CLCG affects pronuclear morphology (OR: 2,65 [1,45–4,85]) and embryo quality (OR: 2,26 [1,25–4,08]) confirmed by several studies [9, 19–21]. Kahraman et al. [14] showed that oocyte CLCG results in 28% of clinical pregnancy with high miscarriage rate of 54% confirming Alikani et al. [22] results. In fact, this negative impact on ART outcomes is evident result of an increased embryonic aneuploidy production risk issued from oocytes with CLCG [14, 16, 23].

However, there are discrepancies in results as reported by Rienzi et al. [3] which could be explained by the presence of intra-individual variation in the percentage of oocytes displaying CLCG in participants of the ICSI program. Nevertheless, Kahraman et al. [14] focused on CLCG phenotype involving its correlation with variables between different couples, either intrinsically or extrinsically linked to environmental conditions. Indeed, oocyte maturation and follicle physiology have been shown to be impaired by persistent environmental pollutants [24]. This issue calls into question the adverse effects of pesticide exposure on oocyte quality, i.e. CLCG prevalence and its developmental ability impacting eventually ART outcomes.

Our ART center is located in Picardy (northern France), a region well known for its high annual pesticide consumption of about 3900 tons used in the agricultural sector. Pesticides and their degradation products can contaminate water, soil, and air; therefore humans can be exposed to these compounds. Moreover, endocrine disruptor chemicals derived from certain pesticides (2,2-Bis [p-hydroxyphenyl]-1,1,1-trichloroethane [HPTE]; antiandrogenic endocrine disruptors; high concentration of total bisphenol A [BPA]; diethylstilbestrol [DES]; methoxychlor [MXC]; polychlorinated biphenyl [PCB] congeners, p,p’-dichlorodiphenyltrichloroethane [DDT], and its persistent metabolite p,p’-dichlorodiphenyldichloroethylene [DDE]) are among the factors that are incriminated for having an adverse impact on several aspects of ovarian biology including oocyte quality [25, 26].

The aim of the present study was to investigate the ICSI outcomes of couples presenting different CLCG prevalence’s of retrieved oocytes (low prevalence of CLCG; fewer than 25% [LCLCG] and high prevalence of CLCG; over 75% [HCLCG]) and its correlation to pesticide exposure zones in the region of Picardy, France.

## Methods

### Patient selection

**Fig. 1:**
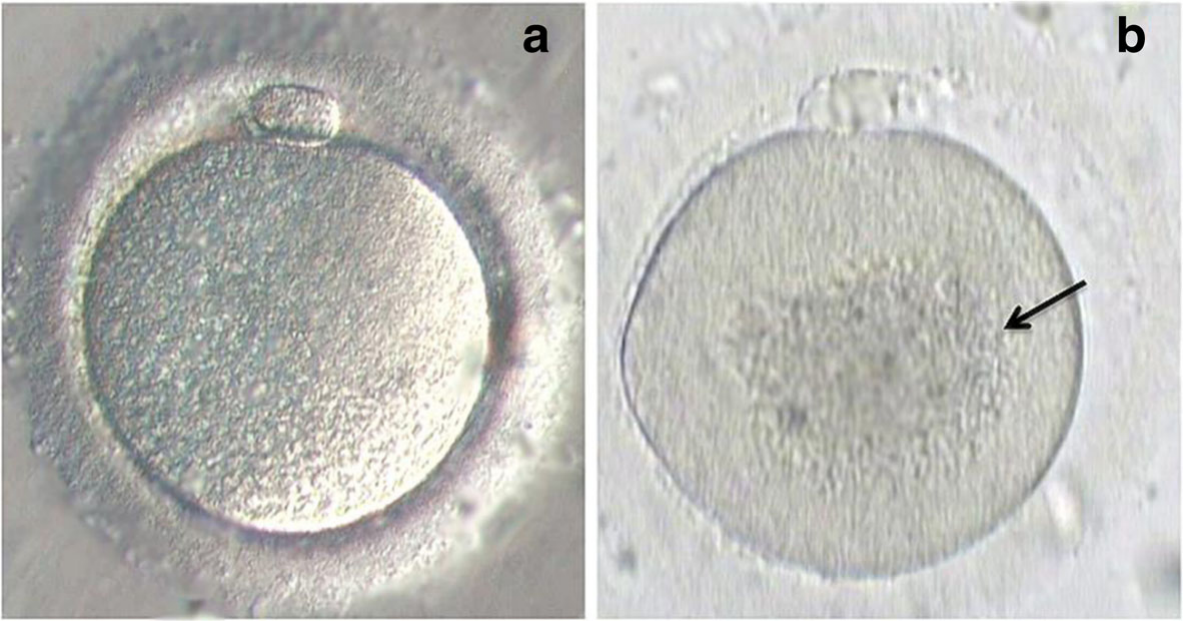
Metaphase II oocytes observed by light microscopy (400× magnification): (**a**) normal oocyte morphology; (**b**) abnormal oocyte exhibiting centrally located cytoplasmic granular area (CLCG; *black arrow*)

The included population in our retrospective study consisted of 633 couples who attended a reproductive medical center and who were part of an ICSI program between 2009 and 2011. Our study population is described in Table 1. In the selected couples, women were normo-responders (oocyte cohort over 5), under 35 years old, living in the Picardy, France region, and exhibited CLCG after oocyte morphology evaluation which was confirmed by a second embryologist (Fig. 1). We excluded couples with severe male infertility factors (severe oligoasthenoteratospermia, azoospermia, or high sperm DNA fragmentation of over 30%) and women aged over 35 in order to minimize the negative impact of female age and/or male factors on oocyte activation and subsequent embryo development [25].

**Table 1:** Baseline characteristics of infertile couples undergoing ICSI and selected for CLCG oocyte evaluation

		LCLCG (*n* = 83)	HCLCG (*n* = 68)	*p*-value
Female characteristics	Age (years)	30 ± 6.4	29 ± 6.4	ns
	Tobacco use (%)	40%	31%	ns
	FSH level at day 3 (IU/ML)	6.1 ± 1.5	6.5 ± 2.3	ns
	E2 level at day 3 (pg/mL)	41.8 ± 12.3	42.9 ± 9.5	ns
	AMH (ng/mL)	2.1 ± 0.4	2.2 ± 0.3	ns
Male characteristics	Age (years)	34 ± 7.8	33 ± 5.5	ns
	Tobacco use (%)	36%	34%	ns
	Sperm concentration (×10^6^/mL)	18 ± 16.5	22.5 ± 20.1	ns
	Sperm motility (%)	26%	30%	ns
	Normal morphology^a^ (%)	23%	28%	ns
Infertility duration (years)		3.4 ± 1.9	3.6 ± 2.7	ns
Secondary infertility (%)		23%	20%	ns
Infertility type	Tubal factor	11%	18%	ns
	Ovulatory factor	24%	16%	ns
	Endometriosis	59%	59%	ns
	Uterine factor	8%	6%	ns
ICSI attempt		2 ± 1.1	2 ± 1.2	ns
COS characteristics	GnRH antagonist use (%)	15%	22%	ns
	COS duration (days)	11.6 ± 2.3	11.2 ± 1.9	ns
	FSH total dose (IU)	1967 ± 935	2219 ± 1203	ns
	E2 level at trigger day (pg/mL)	2760 ± 1142	2627 ± 1076	ns
	Endometrial thickness at trigger day (mm)	10.7 ± 1.8	10.9 ± 2	ns

Results are expressed as *n*(%) or mean (M) ± standard deviation (SD). A difference was considered significant (s) when *P* < 0.05; (ns): not significant

*LCLCG* Low Centrally Located Cytoplasmic Granulation (CLCG) in a group of couples with oocytes exhibiting a low prevalence of CLCG of under 25%, *HCLCG* High CLCG in a group of couples with oocytes exhibiting a high prevalence of CLCG of over 75%, *ICSI* Intracytoplasmic Spermatozoa Injection, *COS* Controlled Ovarian Stimulation, *FSH* Follicle-Stimulating Hormone, *AMH* Anti-Müllerian Hormone, *E2* Estradiol

^a^Spermatozoa were evaluated by the Cohen-Bacrie modified morphology classification (normal forms ≥ 20%)

All selected participants were divided into two groups based on prevalence of CLCG exhibited by total retrieved oocytes. The first group (LCLCG: low CLCG; *n* = 83) included a group of couples with oocytes exhibiting a low prevalence of CLCG under 25%. The second group (HCLCG: high CLCG; *n* = 68) included a group of couples with oocytes exhibiting a high prevalence of CLCG over 75%.The included patients in each group presented unremarkable clinical histories and comparable clinical features as shown in Table 1.

Information was also collected about couples’ location residing in exposed areas considered as the risk zones of pesticide exposure which were evaluated according to the quantity of pesticide-derived active compounds found in surface water (expressed in g/ha of cultivable agricultural land).

### Controlled ovarian stimulation

Couples underwent different controlled ovarian stimulation (COS) protocols based on the patient’s profile and on the physicians’ clinical preference, including long GnRH agonist protocols or antagonist protocols. For long GnRH agonist use, triptorelin was most often administered (Decapeptyl®, Ipsen Pharma, Paris, France) initiated on day 20 of the previous cycle at a dose of 0.1 mg per day, followed 14 days later with half an ampule of triptorelin (0.1 mg per day) combined with FSH (Fostimon®, Genevrier, Sophia-Antipolis, France/Gonal-F®, Merck, Lyon, France/Puregon®, MSD, Levallois-Perret, France) or with hMG (Menopur®, Ferring SAS, St Prex, Switzerland). For antagonist protocols, the stimulation was started from day 2 of the cycle with FSH or hMG, with the initiation of cetrorelix (Cetrotide®, Merck, Lyon, France), 0.25 mg per day when the follicle size exceeded 14 mm or the estradiol (E2) level was over 400 pg/mL. The gonadotropin doses used for COS (FSH or hMG) were adjusted after the first ultrasound examination and E2, LH evaluations on day 7 of the gonadotropin administration. When at least three follicles had reached a diameter ≥ 17 mm, a dose of 250 μg of recombinant human chorionic gonadotropin-rhCG (Ovitrelle®, Merck, Lyon, France) was administered and an oocyte trigger was performed 35 h after hCG administration. Luteal phase supplementation consisted of 400 mg per day of intra-vaginal micronizin progesterone (Utrogestan®; Besins International, Paris, France) from the evening of oocyte retrieval until the β-hCG assay 15 days later.

### ICSI procedure

For the ICSI procedure, the cumulus and corona radiata were removed mechanically under a dissecting microscope and with exposure to 0.5% hyaluronidase (Sigma Company, NY, USA) for 30 s. Sperm from partners was analyzed according to WHOM [27] and treated for ICSI. The treatment was performed after 18 h of incubation at 37 °C in a humidified atmosphere with 5% CO2.Oocytes were examined for the presence of two pronuclei as a sign of fertilization.

The resulting embryos were cultured up to day 3 [28]. Adequate embryo quality (good quality embryos; A + B) was defined based on the presence of uniform size and shape of blastomeres and fragmentation lower or equal to 10% [25]. Fewer than 3 of the best embryos were transferred at day 2 or 3 in utero using a Frydman catheter (CCD Laboratories, Paris, France) and other good quality embryos were cryopreserved.

Clinical pregnancy was confirmed by ultrasound imaging 6–8 weeks after embryo transfer with a β-hCG level above 1000 IU/L and calculated relative to the number of transferred cycles. Ongoing pregnancy was defined as pregnancy with more than 12 weeks of amenorrhea (WA).The miscarriage ratio was calculated relative to the number of clinical pregnancies after the first trimester and was classified as either early (before 12 WA) or late (12 to 20 WA). Each couple went through a single ICSI cycle during this study.

### Statistical analysis

Data are presented as the mean ± standard deviation (SD) or percentage of the total. Data were analyzed with Student’s *t*-test for comparison of mean values or with the chi-squared test for comparison of percentages using Statistical Package, version 6.0 (Statistica). Differences with a value of *p* < 0.05 were considered statistically significant. Multivariate logistic regression analysis was used to test the association between the oocytes and CLCG prevalence and early miscarriages. Odds ratios (ORs) (95% confidence intervals [CI]) were calculated adjusting other involved factors on IVF clinical outcomes and considered significant when *p* < 0.05.

## Results

Couples included in the study and undergoing the ICSI program presenting oocytes with LCLCG or HCLCG showed some differing embryological outcomes, particularly in the embryo cleavage rate (99% vs 82%, respectively) and the number of cryopreserved embryos per patient (1.8 vs 1.2, respectively).The difference in the embryo cryopreservation rate between groups was, however, not significant. In contrast, fertilization and embryo quality rates were not affected by the CLCG degree (Table 2).

**Table 2:** ICSI outcomes in couples compared to CLCG prevalence

		LCLCG (*n* = 83)	HCLCG (*n* = 68)	*p*-value
Total number of oocytes		1178	943	-
Oocytes per patient		14.1 ± 5.7	13.6 ± 5.5	ns
MII per patient		10.4 ± 4.1	9.4 ± 4.2	ns
Maturation rate (%)		(868/1178) 74%	(653/943) 69%	ns
Embryological outcomes	Fertilization rate (%)	(577/868) 66%	(388/653) 59%	ns
	Cleavage rate (%)	(576/577) 99%	(321/388) 82%	0.04 (s)
	Best embryo quality (A + B) rate (%)	(358/576) 62%	(200/321) 62%	ns
	Embryo cryopreservation rate (%)	(150/423) 35%	(78/211) 37%	ns
	Cryopreserved embryos per patient	1.8 ± 0.5	1.2 ± 0.7	0.01 (s)
Total number oftransferredembryos		153	110	-
Transferred embryos per patient		1.9 ± 0.8	1.6 ± 1	ns
Total embryo transfer cycles		79	55	-
Clinical outcomes	Clinical pregnancy rate (%)	(28/79) 35%	(15/55) 27%	ns
	Early miscarriage rate (%)	(3/28) 11%	(7/15) 47%	0.01 (s)
	Ongoing pregnancy rate (%)	(25/79) 32%	(8/55) 14%	0.01 (s)
	Late miscarriage rate (%)	(1/25) 4%	(1/8) 12%	0.02 (s)
	Live birth rate (%)	(24/79) 30%	(7/55) 13%	0.01 (s)

Results are expressed as *n*(%) or mean (M) ± standard deviation (SD). A difference was considered significant (s) when *P* < 0.05; (ns), not significant

*LCLCG* Low Centrally Located Cytoplasmic Granulation (CLCG)in a group of couples with oocytes exhibiting a low prevalence of CLCG of under 25%, *HCLCG* High CLCG in a group of couples with oocytes exhibiting a high prevalence of CLCG of over 75%, *MII* Metaphase II

Clinical outcomes revealed a highly significant difference between LCLCG and HCLCG groups expecting a clinical pregnancy rate. Compared to the LCLCG group, ongoing pregnancy and live birth rates were decreased in couples with HCLCG (32% and 30% vs 14% and 13%, respectively) and early and late miscarriage rates were increased (11% and 4% vs 47% and 12%) (Table 2). Moreover, after multivariate analysis, the OR (95%CI) for early miscarriages in the HCLCG group was 3.1 [2.1–4.1] (*p* < 0.01).

**Fig. 2:**
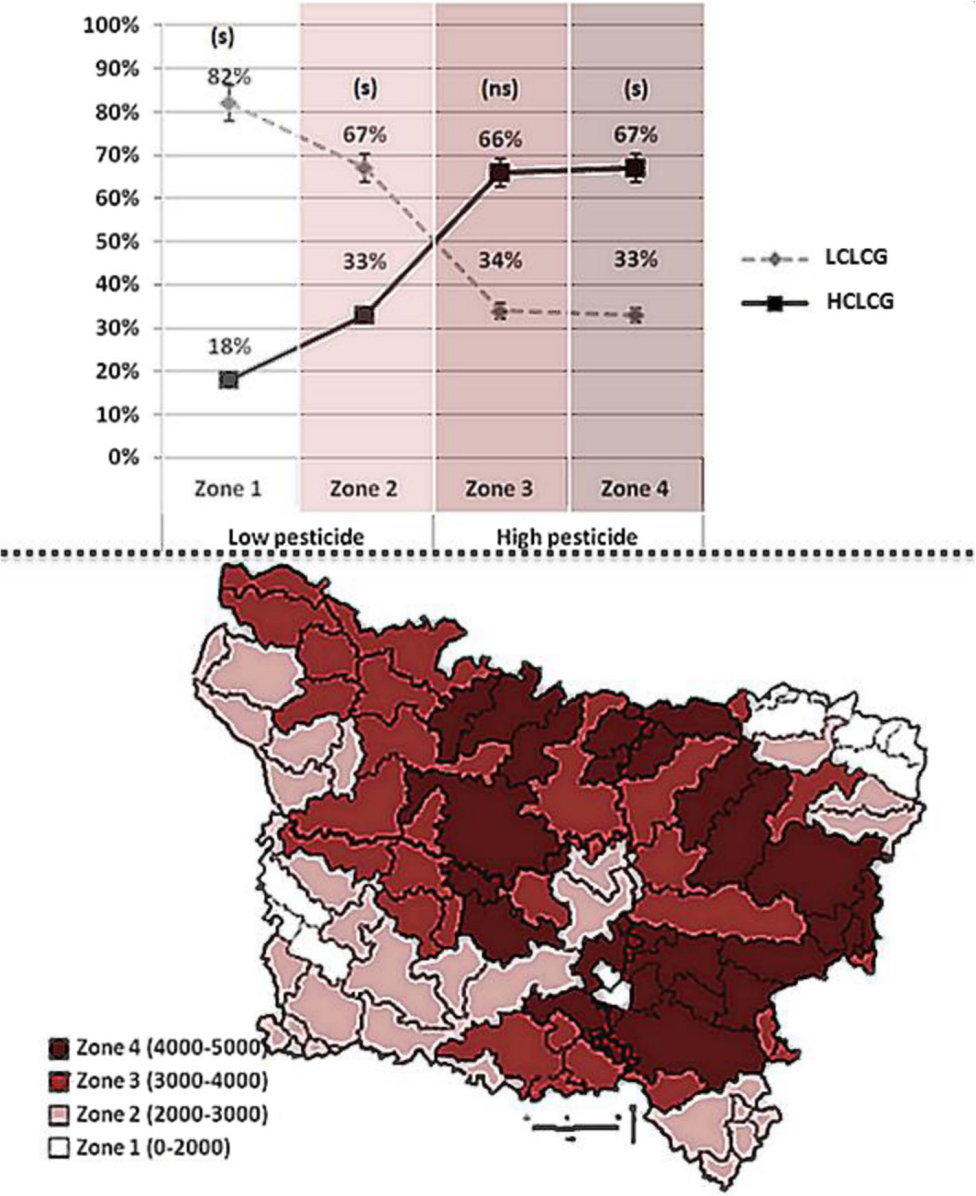
Couples exhibiting LCLCG and HCLCG in different zones of pesticide exposure. LCLCG: Low Centrally Located Cytoplasmic Granulation (CLCG) in a group of couples with oocytes exhibiting a low prevalence of CLCG of under 25%; HCLCG: High CLCG in a group of couples with oocytes exhibiting a high prevalence of CLCG of over 75%. The map of the Picardy region of France for pesticide studies was created in 2009 by GREPP (the Picardy regional group for pesticide studies. The pesticide exposure estimation in each zone was calculated in g/ha in the surface water mass used in the agricultural area. The main zone of low pesticide exposure included zones 1 and 2,and zones 3 and 4 represented the main zone of high pesticide exposure. A difference was considered significant when stage < 0.05; (ns): not significant. Four zones of pesticide use: - Zone 1: 0 to 2000 g/ha (n: 13 areas) – 28 women lived in this zone. - Zone 2: 2000 to 3000 g/ha (n: 29) – 55 women lived in this zone. - Zone 3: 3000 to 4000 g/ha (n: 25) – 32 women lived in this zone. - Zone 4: 4000 to 5000 g/ha (n: 21) – 36 women lived in this zone

In addition, in order to demonstrate a correlation between the location of couples residing in exposed areas with high concentrations of pesticide (to define the risk zone) and degrees of CLCG exhibited by oocytes (LCLCG and HCLCG), Fig. 2 shows a significantly higher number of couples with HCLCG compared to those with LCLCG in risk zones. Zone 4 had a high pesticide exposure over 3000 g/ha, and HCLCG compared to LCLCG was 67% vs 33% (*p* < 0.05). In zones 1 and 2, there was a significantly lower HCLCG compared to LCLCG, but zone 3 did not show a significant difference between groups (Fig. 2).

## Discussion

Since the beginning of implementation of ART to treat human infertility, oocyte quality has always been considered the most important key to IVF success, involving various variables to determine its final ability to produce a normal embryo. One-quarter of oocytes have double anomalies, 6% have triple anomalies, and 3% have abnormal shapes [6, 8–10]. Furthermore, the most damaging oocyte dysmoprhism is that linked to its cytoplasmic immaturity, as in the case of CLCG leading to heterogeneity in the oocyte cohort [14]. This condition is implicitly reflected in the problem of COS response with unbalanced ovarian microenvironment, resulting in a negative impact on IVF outcomes [26].

Several studies have shown that CLCG prevalence varies from 32% to 63% for mature oocytes, showing lack of consensus between several authors about the negative impact of CLCG on fertilization [11, 19], embryo cleavage [29], its quality [8, 14, 19] and its ability to reach blastocyst stage [5]. Nevertheless, almost of studies were more agreed about the negative impact of CLCG on clinical outcomes [3, 5, 6, 8, 11, 14, 29]. In our study, among 633 initial ICSI cycles, 482 couples (76%) had moderate CLCG with a prevalence of 26–74% and were excluded from our study to avoid the non-interpretable results with such a heterogeneous population. For this reason, we decided to focus on analyzing the ICSI outcomes of couples with LCLCG (in 13% of initial cycles [83/633]) and those with HCLCG (in 11% of initial cycles [68/633]), suggesting that CLCG prevalence could have relative impact on ICSI outcomes.

It has been reported that CLCG presence affects the fertilization rate (OR [95%CI]: 1.22 [1.03–1.45]) [30] and Ebner et al. [5] were able to show the association between lower fertilization rate and CLCG. In our study, the negative effect on fertilization rate was not sufficient to be significant between LCLCG and HCLCG (Table 2), while other researchers failed to observe any impact of CLCG on fertilization rates [3, 4, 14, 20, 22, 27, 31–33]. This issue could be explained by the ability of endoplasmic reticulum in the oocyte to composite the intrinsic dysregulation by stimulating calcium flux after injection of spermatozoa by ICSI and assuring the necessary activation for fertilization despite CLCG presence [34–36], whereas oocytes with aggregation of the endoplasmic reticulum were associated with a lower fertilization rate and poor embryo quality [29, 31].

However, the correlation between CLCG exhibited by oocytes and their impaired developmental competence has been discussed in detail by many authors [6, 37, 38]. These works confirm our results regarding embryo cleavage rate (99% for LCLCG vs 82% for HCLCG) (Table 2). Nevertheless, discrepancies between studies that show a difference in cleavage rates could be explained by the involved other factors on embryo cleavage decrease. Among them, we can cite in one hand the lack of rigorous oocyte morphology evaluation including other possible less apparent of oocyte dysmorphisms. In the other hand, there is risk of sperm genome decays presence on the selected spermatozoa for ICSI which is morphologically normal [25].

Moreover, Ebner et al. [5] proved the negative correlation between CLCG and blastulation rate (44%). Contrary to previous findings on embryo cleavage rate, some authors did not find lowered in vitro developmental ability in oocytes with CLCG compared to those with complete absence of granulation in the cytoplasm [14, 20, 29, 32]. When other cytoplasmic features were compared to CLCG, the assessment of their predictive value showed less consistent results, whereas CLCG presence showed the strongest association with a value of f 2.7 presenting the major point in the Rienzi’s score (Metaphase II oocyte morphological scoring system MOMS) [3]. The negative impact of CLCG on embryonic morphology could not be significantly demonstrated [4]. It could not be proved for the embryo quality rate either, with a risk of 1.15 [0.56–2.36] [33]. Generally, oocyte morphology could affect embryo cleavage contrarily to embryo quality. There was no real significant difference between abnormal extra-cytoplasmic or cytoplasmic morphological features in oocytes [39–41]. This could explain why we found a significant difference in embryo cleavage rate and not in embryo quality rate (Table 2). With more available embryos, it was easier to obtain more cryopreserved embryos from an oocyte cohort with a CLCG under 25% (two embryos per patient) than those with a CLCG over 75% (one embryo per patient) (Table 2). Nevertheless, Balaban et al. [37] reported that embryos derived from oocytes with CLCG had decreased survival rate and impaired in vitro development after cryopreservation. Hence, the cryopreservation of embryos issued from dysmorphic oocytes need to be studied, perhaps using a genetic evaluation and aneuploidy testing is still unclear and has sparked a debate among authors [14, 15, 18, 32, 42, 43].

With a selection based only on morphological features from MII oocytes until the embryo transfer stage, seemingly appropriate and normal but intrinsically the impaired embryos might be transferred, resulting in poor clinical outcomes [16]. Balaban and Urman [29] did not find any effect comparing all oocyte anomalies with miscarriage rates (20% for abnormal oocytes vs 14% for normal oocytes) nor with pregnancy rates in CLCG compared to a control group (39% and 42%, respectively). However, most oocytes with an abnormal morphology have been shown to be associated with a poor implantation or pregnancy rate [6, 8, 9, 14, 19, 32, 34, 39]. Indeed, significantly lower pregnancy rates were reported with oocytes with cytoplasmic anomalies (3% for abnormal oocytes vs 24% for normal oocytes) [44]. In the present study, we were able to demonstrate a clear negative impact of HCLCG compared to LCLG on clinical outcomes including ongoing pregnancy and live birth rates which were decreased in couples with HCLCG (32% and 30% vs 14% and 13%, respectively) (Table 2), as confirmed by the study of Kahraman et al. [14]. Nevertheless, though the pregnancy rate was affected by CLCG presence as reported by some authors [6, 14, 23]; the difference in pregnancy rate between groups in the present work was not significant. Wilding et al. [20] also reported these findings, demonstrating the need to calculate the study power with more consistent study populations. However, this result could be explained by a possible presence of embryonic aneuploidies [14, 16, 18, 25, 34, 35] issued from oocytes with CLCG which are responsible for implantation failures before clinical pregnancy. There is probable involvement of other intra-uterine dysregulating factors [28] which are associated with oocyte dysmorphism [11, 13, 35].

Early miscarriage rates in our study were increased from 11% for LCLCG to 47% for HCLCG, and this result was confirmed after multivariate analysis, resulting in a risk of 3.1 (2.1–4.1) when CLCG was over 75% (Table 2). This topic was previously elucidated by Otsuki et al. [11] and Ebner et al. [5], highlighting the association between CLCG and other cytoplasmic organelle dysregulations involving the endoplasmic reticulum and the mitochondria with high levels of oxidative stress and division spindle anomalies [11, 14, 35]. This condition could produce aneuploidies [1, 11, 14, 34] and could very well explain why Otsuki et al. [11] could report the case of a newborn with Beckwith-Wiedemann syndrome, a genetic imprinting disorder affecting chromosome 11p15.5, associating it with oocyte dysmorphism with sERC. They suggested that it was linked to CLCG production in oocytes whereas the endoplasmic reticulum in the oocyte stores and redistributes calcium, enabling cell activation during fertilization and energy accumulation by mitochondria necessary for appropriate embryo cleavage and an eventual maintained pregnancy [34–36]. Although there is no clear difference between oocyte dysmorphisms based on oocyte morphology evaluation, other investigations are needed to explore further associations between each oocyte morphologic anomaly and aneuploidy affecting clinical outcomes as well as epigenetic modifications with a risk of impairment of offspring [45].

Although several authors are debating the effect of oocyte dysmorphism on clinical outcomes [6, 8, 9, 19–22], we cannot neglect the importance of oocyte morphology evaluation particularly when it is directly correlated to the negative effect of toxic environments.

Indeed, a large proportion of human oocytes resulting from exogenous gonadotropin-stimulated cycles have different morphological attributes or dysmorphisms [12, 42]. Conversely, other authors did not find negative effects of COS protocol on oocyte quality and IVF outcomes [3, 7, 39] involving other factors such as female age [46], AMH, total FSH dose, COS duration [5], or estradiol level [47].

Apart from the toxic intra-ovarian environment produced intrinsically by COS with exogenous gonadotropins, the developing ovarian organ is particularly sensitive to environmental harm caused by pesticide exposure affecting oocyte quality [48].

Some pesticides, (e.g. dichlorodiphenyltrichloroethane [DDT], methoxychlor, DES, and vinclozolin) are endocrine disruptors with estrogen-like effects. In women, exposure to bisphenol A affects folliculogenesis (via the granulosa and theca cells [49]), induces meiotic aberrations (aneuploidy), and leads to a decreased oocyte quality during IVF.

In contrast, diethylstilbestrol (DES), which represents a well-studied estrogenic chemical, was proved in murine model to cause oocyte dysmorphism with condensed chromatin along the nuclear membrane [45], ovarian cysts, and ovarian tumors [50]. DDE, the most stable metabolite of DDT, and vinclozolin are persistent organic pollutants in follicular fluid, targeting androgen receptors. They stimulate aromatase and act in synergy with FSH to induce a premature rise in estradiol levels, affecting oocyte maturation. In addition, they have been found to induce the intra-ovarian inflammatory process involving VEGF and IGF-1; negatively affecting intra-ovarian mechanisms [51–54]. Moreover, methoxychlor (MXC), an organochlorine pesticide, has had anti-estrogen activity in the ovary [55], inhibiting growth and inducing atresia of antral follicles through an oxidative stress pathway with an effect on AMH production [54].

It is known that the fetus is particularly vulnerable to pesticide exposure because of its rapid growth, the sensitivity of its developing organs, and the immaturity of its metabolic pathways and enzymatic defenses. A Canadian study described an association between pesticide exposure and increased miscarriage rate [56], contrary to Willis et al. [57] in a Californian study. In our study focusing on the region of Picardy, France, pesticide exposure zones were relatively correlated to CLCG prevalence as the risk zone having the highest pesticide exposure over 3000 g/ha was the residence place of 67% of couples with HCLCG compared to 33% of couples with LCLCG (Fig. 2).

The limitations of our study relate to our lack of data on the type of pesticides used (because one can legitimately consider that different types of products have different effects), the impact of meteorological and hydrological conditions which can modify the pesticide profile, and the type of exposure (acute or chronic). Moreover, despite this study was focused on evaluation of the correlation presence between residence couples included in risk zones of pesticides exposure and CLCG, this issue cannot prove the exposure occurrence especially that duration of residence was unknown. Nevertheless, this study could to demonstrate a relative correlation between pesticide exposure and risk of CLCG and the extension of this study will be the evaluation of different pesticide concentrations in serum and follicular fluid to establish the causality of certain compounds on oocyte quality, embryonic development, and clinical outcomes.

## Conclusion

Further special attention paid to oocyte morphology evaluation with cytoplasmic attributes, including CLCG among other oocyte dysmorphisms, could indirectly reflect a dysregulated intra-ovarian microenvironment involving various variables and impaired oocyte maturation affecting ICSI clinical outcomes. The high prevalence of CLCG exhibited by the oocyte cohort over 75% might be a predictive factor for early miscarriage risk. In addition, risk zones with high pesticide exposure demonstrated more risk of oocytes with a high prevalence of CLCG. There is a real need for investigating pesticide components and their associations with oocyte dysmorphism, including CLCG features, aneuploidy evaluation, and ICSI outcomes.

## Abbreviations

ART: Assisted reproductive technologies
aSERT: smooth endoplasmic reticulum
BPA: antiandrogenic endocrine disruptors; bisphenol A
CLCG: Centrally located cytoplasmic granulation
COS: Controlled ovarian stimulation
DDE: 1,1-dichloro-2,2-bis(p-chlorophenyl)ethylene
DES: Diethylstilbestrol
E2: Estradiol
HCLCG: High prevalence of CLCG
HPTE: 2,2-Bis [p-hydroxyphenyl]- 1,1,1-trichloroethane
LCLCG: Low prevalence of CLCG
MII: Metaphase II
MXC: Methoxychlor
sERC: Smooth endoplasmic reticulum clusters
WA: Weeks of amenorrhea
